# Plasma carbohydrate antigen-125 for prediction of atrial fibrillation recurrence after radiofrequency catheter ablation

**DOI:** 10.1186/s12872-021-02207-y

**Published:** 2021-08-19

**Authors:** Qingya Wang, Chengjing Dang, Haoyu Liu, Jie Hui

**Affiliations:** 1 Department of Cardiology, The First Affiliated Hospital of Soochow University, Suzhou, 215006 China; 2 Department of Physical Medicine and Rehabilitation, The First Affiliated Hospital of Soochow University, Suzhou, 215006 China

**Keywords:** Atrial fibrillation, Carbohydrate antigen-125, Radiofrequency catheter ablation, Recurrence

## Abstract

**Background:**

Elevated plasma carbohydrate antigen-125 (CA-125) levels are strongly associated with new-onset atrial fibrillation (AF) and heart failure, but the relationship between plasma CA-125 level and AF recurrence following radiofrequency catheter ablation (RFCA) remains poorly investigated. We aimed to assess whether elevated CA-125 levels are related to long-term AF recurrence following RFCA.

**Methods:**

Preoperative CA-125 levels were determined in AF patients undergoing initial RFCA. Multivariate-adjusted Cox models were constructed to determine the relationship between CA-125 levels and AF recurrence. Multivariate logistic regression analyses were performed to determine predictors of AF recurrence.

**Results:**

Of the 353 enrolled patients, 85 patients (24.1%) had AF recurrence at the 12-month follow-up. These patients had significantly higher baseline CA-125 levels than those without AF recurrence [(18.71 ± 12.63) vs. (11.27 ± 5.40) U/mL, *P* < 0.001]. The incidence of AF recurrence across quartiles 1–4 of CA-125 was 11.5%, 13.3%, 21.6% and 50.0%, respectively (*P*-trend < 0.001). The adjusted hazard ratios (aHRs) for AF recurrence across quartiles 1–4 of CA-125 were 1.00 (reference), 1.085 (95% CI, 0.468–2.520), 1.866 (95% CI, 0.867–4.019), and 4.246 (95% CI, 2.113–8.533), respectively (*P*-trend < 0.001). A similar effect was obtained when CA-125 was studied as continuous data (aHR per unit increase in LnCA-125, 3.225, 95% CI, 2.258–4.606; *P* < 0.001). When a predefined CA-125 cut-off of 13.75 U/mL was established, patients with CA-125 ≥ 13.75 U/mL had a higher risk of recurrent AF than those with CA-125 < 13.75 U/mL (aHR, 3.540, 95% CI, 2.268–5.525, *P* < 0.001). Multivariate analysis revealed CA-125, high-sensitivity C-reactive protein, and left atrium anteroposterior diameter as independent risk factors for AF recurrence.

**Conclusions:**

Elevated preoperative CA-125 levels are related to a higher risk of AF recurrence and can independently predict AF recurrence following RFCA.

## Background

Atrial fibrillation (AF) is one of the most common arrhythmias in clinical practice. In 2010, an estimated 33.5 million people globally were diagnosed with AF, including approximately 20.9 million men and 12.6 million women [1]. The prevalence of AF increases with age; it is approximately 1% in people younger than 60 years, rising to approximately 12% in people aged 75–84 years, and over 33% in people aged 80 years and older [2]. AF commonly leads to heart failure (HF), stroke, and sudden cardiac death, and is increasing the burden on healthcare due to an aging global population and increased co-morbid survival rates [1]. With the increasing effectiveness and safety of radiofrequency catheter ablation (RFCA), the procedure has been widely used in the clinical treatment of AF as a Class I recommendation in recent years [3, 4]. However, it is not possible for all patients to maintain sinus rhythm after the procedure, and the reported recurrence rate of AF is 20–45% following the ablation [5]. The high rate of recurrence after RFCA remains a concern for clinicians. Indicators that can effectively and accurately predict recurrent AF following RFCA will facilitate the selection of patients who will most benefit from this procedure. Moreover, the use of such indicators will improve the success of the operation and guide clinical practice.

Various biochemical and clinical predictors of AF recurrence have been identified previously. Among these predictors are left atrium anteroposterior diameter (LAD), persistent AF (PeAF), age, HF, diabetes, hypertension, thromboembolic scores (CHADS2 and CHA2DS2-VASc scores), atrial fibrosis (analyzed by magnetic resonance imaging), and biomarkers, such as high-sensitivity C-reactive protein (hs-CRP) and N-terminal pro-brain natriuretic peptide [6, 7]. However, differences remain across studies, and additional risk factors associated with AF recurrence have not been thoroughly investigated.

Carbohydrate antigen-125 (CA-125) has traditionally been considered a biomarker for monitoring ovarian cancer therapy, and a serum value < 35 U/mL is considered normal [8]. It is a high-molecular-weight soluble glycoprotein commonly expressed in the coelomic epithelium, such as pericardium, pleura, peritoneum, and Müllerian epithelium, and is released into the circulation when these tissues are stimulated by mechanical stress or inflammation, exhibiting a high expression state [9]. In recent years, elevated CA-125 levels in cardiac diseases, such as acute and chronic HF, pericarditis, coronary atherosclerotic heart disease, and AF have been reported in many studies, and its relevance in the prognosis of these diseases has been studied [10–14]. It is used in cardiac diseases primarily to monitor congestion and inflammation and has recently emerged as a potential replacement for fluid retention and inflammatory activation in acute and chronic HF [15]. However, almost no studies have reported the relationship between CA-125 and the late recurrence of AF after RFCA. The present study aimed to investigate the relationship between elevated pre-interventional CA-125 levels and AF recurrence during a follow-up of 12 months.

## Methods

### Study population

**Fig. 1:**
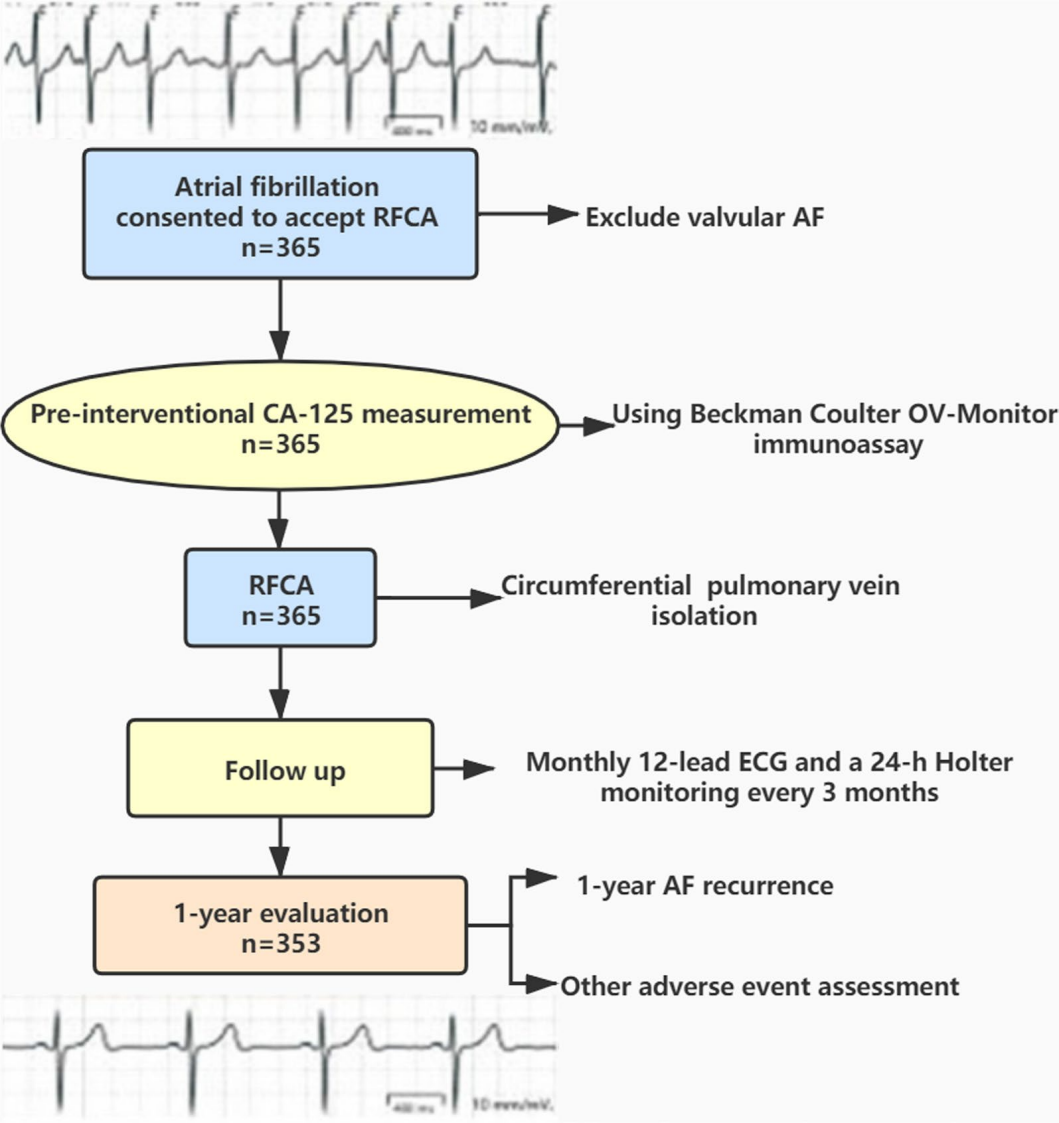
Flow chart of patient selection and CA-125 measurement. RFCA, radiofrequency catheter ablation; AF, atrial fibrillation; CA-125, carbohydrate antigen-125; ECG, electrocardiogram

The study population of this prospective cohort study comprised AF patients who underwent their initial RFCA at the Department of Cardiology of the First Affiliated Hospital of Soochow University between February 2017 and February 2020. All patients were followed up for 12 months following ablation. The inclusion criteria were as follows: non-valvular AF; > 18 years of age; suitable for RFCA and voluntary participation in this study; RFCA treatment for the first time; and signed informed consent. The exclusion criteria were as follows: history or findings of cardiovascular disease, including rheumatic heart diseases, pericarditis, coronary atherosclerotic heart disease, and HF symptoms or systolic dysfunction [left ventricular ejection fraction (LVEF) < 50%]; severe hepatic and/or renal dysfunction; acute and chronic inflammatory diseases; autoimmune diseases; undergoing treatment with steroids and/or immunosuppressants; neoplastic diseases, especially ovarian cancer; serous cavity effusion; a high CA-125 level of 100 U/mL (suspected malignancy); death or loss of follow-up. The patient selection flowchart is shown in Fig. 1.

The study was performed in accordance with the Declaration of Helsinki for Human Research and was approved by the Medical Ethics Committee of the First Affiliated Hospital of Soochow University.

### Data collection

Data on baseline characteristics of AF patients at admission were collected from the hospital medical records, including: (1) general clinical data: age, sex, body mass index (BMI), systolic blood pressure, diastolic blood pressure, duration of AF, comorbidities and calculation of CHADS2 score, CHA2DS2-VASc score, ablation procedures, and history of medication; (2) hematological indices (results of fasting blood sample obtained on the latest preoperative morning): hemoglobin, low-density lipoprotein cholesterol, high-density lipoprotein cholesterol, triglyceride, fasting plasma glucose, serum creatinine, uric acid, and high-sensitivity C-reactive protein (hs-CRP); echocardiographic parameters (preoperative measurements were performed with a Philips IE33 color Doppler ultrasound): LAD, left ventricular end-diastolic diameter, left ventricular end-systolic diameter, and LVEF.

### Blood sampling

Fasting peripheral venous blood samples were collected and processed on the day before ablation. Plasma CA-125 levels were determined using Beckman Coulter OV-Monitor immunoassay strictly following the manufacturer's instructions, with the levels expressed as U/mL.

### Catheter ablation

All antiarrhythmic drugs except for amiodarone were discontinued for ≥ 5 half-lives before the RFCA. Transesophageal echocardiography was performed to confirm the absence of atrial thrombi on the day of the RFCA. Complete circumferential pulmonary vein isolation (CPVI) was performed in all patients using an irrigated radiofrequency ablation catheter with the aid of electroanatomic mapping (Carto 3; Biosense Webster, USA). Linear ablation in the left atrial roof, superior vena cava isolation ablation, cavo-tricuspid isthmus ablation, mitral isthmus ablation, and complex fractionated electrograms ablation were performed at the discretion of the type of AF and the presence or absence of atrial flutter and atrial tachycardia preoperatively and intraoperatively. After complete CPVI, a minimum waiting time of 30 min from the last CPVI was mandated. Successful ablation was defined as the complete elimination of all fragmented signals at the PV ostium, and the exit and entrance block was verified. If AF persisted after initial ablation, direct current cardioversion was performed to restore sinus rhythm. The use of oral anticoagulants was continued for 3 months postoperatively, and the decision to discontinue oral anticoagulants was made after 3 months at the discretion of the doctor based on the CHA2DS2-VASc score. Whether or not to take antiarrhythmic drugs depended on the specific circumstances after RFCA. Proton-pump inhibitors were administered orally at 2–4 weeks postoperatively.

### Follow-up

After discharge, all patients underwent routine follow‐up at our cardiology clinic where at least a monthly 12-lead ECG and a 24-h Holter monitoring every 3 months were obtained. They were strongly recommended to visit the nearest hospital for an ECG if they felt symptoms that could be attributed to an arrhythmia or noticed any irregularity of their peripheral pulse by routine self-measurement. AF recurrence was defined as any documented atrial tachyarrhythmia (AF, atrial flutter, or atrial tachycardia) episode lasting for at least 30 s after ablation, excluding a 3-month blanking period.

### Statistical analysis

Baseline characteristics of participants were grouped according to the 12-month follow-up results and stratified based on quartiles of CA-125 levels. All continuous data are presented as mean ± SD, and categorical data are presented as count (percentage). One-way ANOVA or Student's *t*-test was used to identify differences in normally distributed continuous data. The Kruskal–Wallis *H*-test or Mann–Whitney *U*-test was used to identify differences in continuous data with a skewed distribution. The chi-square or Fisher's exact test was used to compare categorical data. Correlation was evaluated by the Spearman correlation test.

We employed a multi-step process to investigate the effect of CA-125 levels on the recurrence of AF following ablation. First, patients with AF were classified into four strata according to the quartiles of CA-125 levels. We defined the first quartile as reference. We calculated the rates of recurrence stratified by CA-125 quartiles at 12-month follow-up and compared them using a trend test of the survival function. The cumulative incidence of AF-free recurrence between CA-125 quartiles was estimated using Kaplan–Meier survival curves that were compared using Log-rank tests. The Cox proportional hazards model was constructed to evaluate the AF recurrence risk at CA-125 quartiles. We did not adjust the first model, but we adjusted the second model for sex, age, and BMI and the third model for sex, age, BMI, AF duration in months, type of AF (paroxysmal vs. persistent), history of diabetes (yes vs. no), history of hypertension (yes vs. no), history of dyslipidemia (yes vs. no), intraoperative direct current cardioversion (yes vs. no), ablation procedures, LAD, and hs-CRP. Second, CA-125 levels were log-transformed, and the analysis process was repeated using CA-125 level as continuous data. As described above, we used the same set of variables to adjust for the multivariate models.

For further analysis, the participants were stratified at a CA-125 cut-off point of 13.75 U/mL, which was the best cut-off value for the prediction of AF recurrence in this study. We assessed whether patients with less than or greater than (or equal to) this value had different AF recurrence rates and constructed the same multivariate models as mentioned above.

We used univariate and multivariate logistic regression analyses to define risk factors for recurrence of AF. The correlation variables found to be statistically significant in univariate analyses were identified as independent factors in multivariate analyses. The receiver operating characteristic (ROC) curve analysis was performed to determine the sensitivity and specificity in predicting recurrent AF by CA-125. The area under the curve (AUC) was calculated to assess testing accuracy.

All statistical analyses were completed using SPSS software version 21.0, and a two-sided *P*-value < 0.05 was deemed to indicate a statistically significant difference.

## Results

### Patient characteristics

**Fig. 2:**
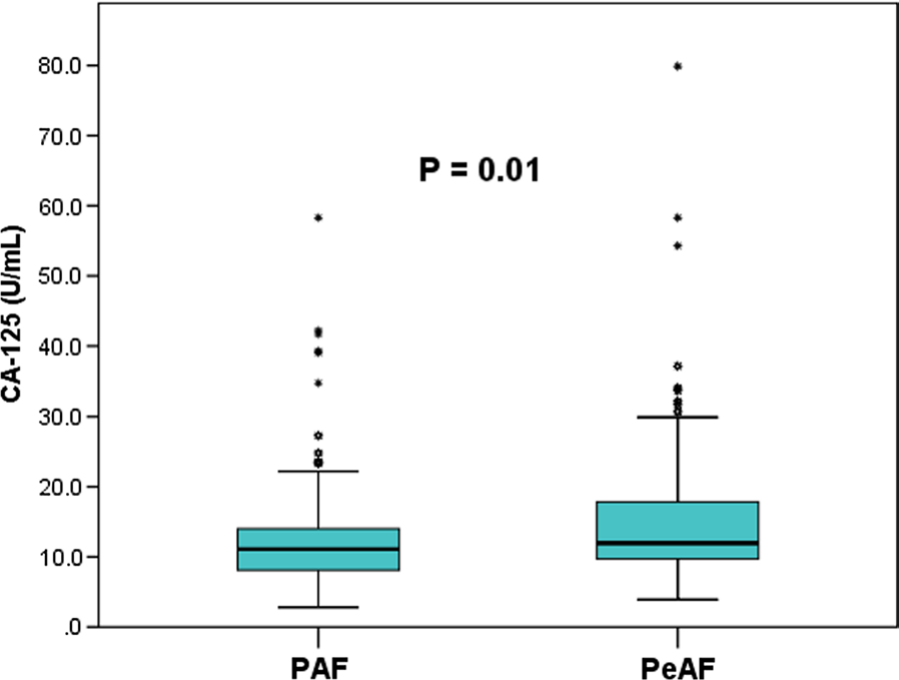
Box plot representing preoperative CA-125 levels in PeAF (right) and PAF groups (left)

**Fig. 3:**
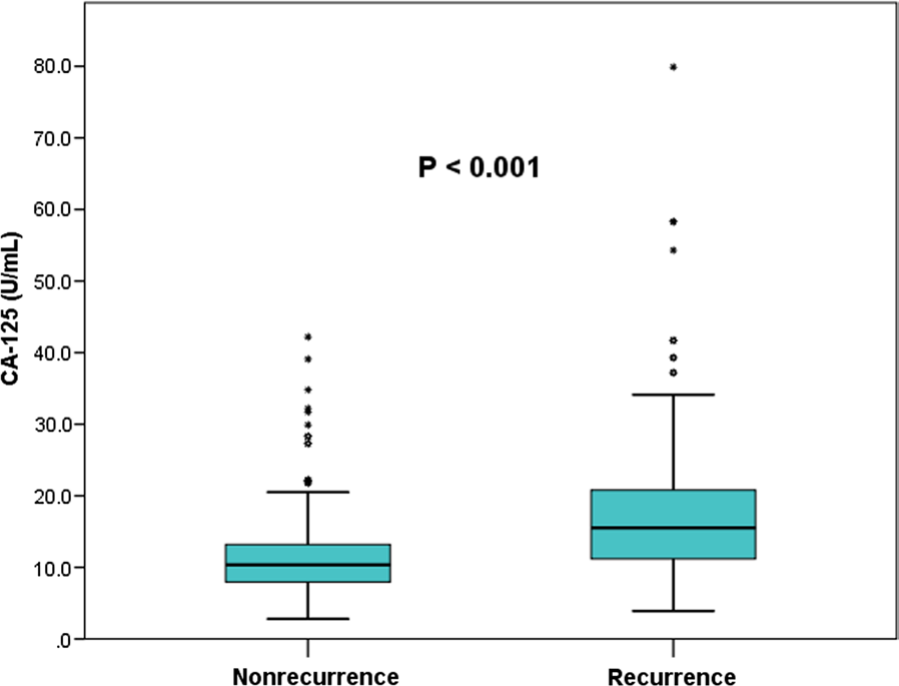
Box plot representing preoperative CA-125 levels in the recurrence (right) and nonrecurrence groups (left)

We enrolled 365 consecutive AF patients in the study, of whom 12 did not complete follow-up, thus making the total of 353 patients for the analysis (Table 1). Of those, there were 96 (27.2%) with PeAF and 257 (72.8%) with PAF. PeAF patients had significantly higher baseline CA-125 levels than PAF patients (15.59 ± 11.59 U/mL vs. 12.12 ± 6.59 U/mL, *P* = 0.01) (Fig. 2). Of the 353 patients, 85 (24.1%) had recurrence of AF during the 12-month follow-up following RFCA, and 15 (4.2%) received a second ablation procedure due to AF recurrence. Patients with recurrent AF had significantly higher baseline CA-125 levels than those without recurrence (18.71 ± 12.63 U/mL vs. 11.27 ± 5.40 U/mL, *P* < 0.001) (Fig. 3; Table 1). Furthermore, significant differences were also observed for AF type, LAD, hs-CRP levels, duration of AF, and frequency of intraoperative direct current cardioversion.

**Table 1:** Characteristics of patients according to AF recurrence vs nonrecurrence

Characteristics	Total	Recurrence	No recurrence	*P*-value
	(n = 353)	(n = 85)	(n = 268)	
*General clinical characteristic*				
Age (years)	61.56 ± 9.47	61.87 ± 9.49	61.46 ± 9.47	0.643
Sex				0.306
Male	199 (56.4)	52 (61.2)	147 (54.9)	
Female	154 (43.6)	33 (38.8)	121 (45.1)	
BMI (kg/m^2^)	24.79 ± 3.09	24.83 ± 3.14	24.77 ± 3.08	0.867
SBP (mmHg)	128.10 ± 15.37	127.94 ± 14.46	128.15 ± 15.68	0.912
DBP (mmHg)	79.18 ± 10.33	79.78 ± 9.93	78.99 ± 10.47	0.541
Type of AF				< 0.001
Paroxysmal	257 (72.8)	48 (56.5)	209 (78.0)	
Persistent	96 (27.2)	37 (43.5)	59 (22.0)	
AF duration (months)	25.26 ± 36.68	29.12 ± 33.32	24.03 ± 37.66	0.045
CHADS2 score	0.90 ± 0.93	1.04 ± 1.06	0.86 ± 0.88	0.288
CHA2DS2-VASc score	1.78 ± 1.37	1.89 ± 1.47	1.75 ± 1.34	0.443
*Comorbidity*				
Hypertension	208 (58.9)	53 (62.4)	155 (57.8)	0.461
Diabetes	34 (9.6)	12 (14.1)	22 (8.2)	0.108
Dyslipidemia	18 (5.1)	6 (7.1)	12 (4.5)	0.509
Stroke/TIA	21 (5.9)	6 (7.1)	15 (5.6)	0.620
*Hematological index*				
Hemoglobin (g/L)	138.70 ± 16.19	140.24 ± 15.02	138.21 ± 16.54	0.316
FBG (mmol/L)	5.07 ± 0.82	5.13 ± 0.99	5.06 ± 0.76	0.925
Sc (μmol/L)	70.10 ± 15.60	73.94 ± 20.37	68.88 ± 13.55	0.163
UA (μmol/L)	347.16 ± 88.31	355.63 ± 92.40	344.46 ± 86.97	0.311
ALB (g/L)	42.32 ± 3.43	42.48 ± 3.89	42.28 ± 3.28	0.637
TC (mmol/L)	4.41 ± 0.95	4.26 ± 1.05	4.45 ± 0.91	0.111
TG (mmol/L)	1.62 ± 1.09	1.71 ± 1.11	1.59 ± 1.08	0.252
LDL-C (mmol/L)	2.56 ± 0.83	2.40 ± 0.80	2.61 ± 0.83	0.126
HDL-C (mmol/L)	1.15 ± 0.29	1.12 ± 0.32	1.16 ± 0.28	0.177
*Echocardiographic parameter*				
LAD (mm)	42.48 ± 5.50	44.68 ± 5.18	41.77 ± 5.42	< 0.001
LVEF (%)	62.84 ± 5.48	61.92 ± 5.14	63.13 ± 5.56	0.076
LVEDD (mm)	48.72 ± 4.46	49.02 ± 5.01	48.63 ± 4.27	0.510
LVESD (mm)	32.03 ± 4.01	32.40 ± 3.97	31.91 ± 4.02	0.331
Ablation procedure				0.453
CPVI alone	232 (65.7)	53 (62.4)	179 (66.8)	
CPVI plus additional ablation	121 (34.3)	32 (37.6)	89 (33.2)	
Direct current cardioversion	103 (29.2)	37 (43.5)	66 (24.6)	0.001
*Medication*				
Oral anticoagulant				0.295
Warfarin	25 (7.1)	9 (10.6)	16 (6.0)	
Dabigatran	255 (72.2)	61 (71.8)	194 (72.4)	
Rivaroxaban	73 (20.7)	15 (17.6)	58 (21.6)	
ACEI/ARB	145 (41.1)	41 (48.2)	104 (38.8)	0.124
β-Blocker	165 (46.7)	45 (52.9)	120 (44.8)	0.189
Amiodarone	249 (70.5)	62 (72.9)	187 (69.8)	0.577
Propafenone	52 (14.7)	15 (17.6)	37 (13.8)	0.384
*Biomarkers*				
hs-CRP (mg/L)	1.97 ± 2.58	2.76 ± 3.45	1.71 ± 2.18	0.004
CA-125 (U/mL)	13.06 ± 8.38	18.71 ± 12.63	11.27 ± 5.40	< 0.001

All continuous variables are expressed as mean ± SD, and categorical variables are presented as count (percentage). BMI, body mass index; SBP, systolic blood pressure; DBP, diastolic blood pressure; AF, atrial fibrillation; TIA, transient ischemic attack; FBG, fasting plasma glucose; Sc, serum creatinine; UA, uric acid; ALB, serum albumin; TC, total cholesterol; TG, triglyceride; LDL-C, low-density lipoprotein cholesterol; HDL-C, high-density lipoprotein cholesterol; ACEI, angiotensin-converting enzyme inhibitor; ARB, angiotensin receptor blocker; LAD, left atrium anteroposterior diameter; LVEDD, left ventricular end-diastolic diameter; LVESD, left ventricular end-systolic diameter; LVEF, left ventricular ejection fraction; hs-CRP, high-sensitivity C-reactive protein; CA-125, carbohydrate antigen-125.

Table 2 presents the characteristics of AF patients at baseline based on CA-125 quartiles. There were significant differences in hs-CRP levels, LAD in the baseline echocardiogram, and AF duration in months across CA-125 quartiles. Correlation analysis showed that CA-125 levels are positively correlated with LAD (r = 0.144, *P* = 0.007) and AF duration (r = 0.136, *P* = 0.011), negatively correlated with LVEF (r =  − 0.108, *P* = 0.044), and not significantly correlated with hs-CRP (r = 0.073, *P* = 0.172).

**Table 2:** Baseline characteristics based on CA-125 quartiles

Characteristics	CA-125 level				*P*-value
	Quartile 1	Quartile 2	Quartile 3	Quartile 4	
	(n = 87)	(n = 90)	(n = 88)	(n = 88)	
*General clinical characteristics*					
Age (years)	62.23 ± 8.87	61.78 ± 9.20	60.14 ± 10.58	62.08 ± 9.13	0.469
Sex					0.957
Male	51 (58.6)	51 (56.7)	48 (54.5)	49 (55.7)	
Female	36 (41.4)	39 (43.3)	40 (45.5)	39 (44.3)	
BMI (kg/m^2^)	24.81 ± 3.10	24.86 ± 2.99	25.12 ± 3.34	24.33 ± 2.93	0.395
SBP (mmHg)	127.79 ± 14.82	131.12 ± 15.46	127.02 ± 16.10	126.40 ± 14.89	0.170
DBP (mmHg)	78.34 ± 10.61	79.82 ± 10.42	80.30 ± 9.69	78.23 ± 10.61	0.443
Type of AF					0.085
Paroxysmal	69 (79.3)	64 (71.1)	68 (77.3)	56 (63.6)	
Persistent	18 (20.7)	26 (28.9)	20 (22.7)	32 (36.4)	
AF duration (months)	20.67 ± 43.62	21.56 ± 31.52	27.66 ± 32.84	31.17 ± 37.31	0.044
CHADS2 score	0.89 ± 0.81	0.82 ± 0.97	0.91 ± 0.78	1.00 ± 1.12	0.587
CHA2DS2-VASc score	1.77 ± 1.15	1.66 ± 1.42	1.76 ± 1.25	1.94 ± 1.61	0.629
*Comorbidity*					
Hypertension	50 (57.5)	48 (53.3)	59 (67.0)	51 (58.0)	0.299
Diabetes	8 (9.2)	8 (8.9)	6 (6.8)	12 (13.6)	0.476
Dyslipidemia	3 (3.4)	1 (1.1)	7 (8.0)	7 (8.0)	0.073
Stroke/TIA	7 (8.0)	5 (5.6)	3 (3.4)	6 (6.8)	0.606
*Hematological index*					
Hb (g/L)	137.67 ± 16.81	137.87 ± 16.19	138.90 ± 15.71	140.35 ± 16.18	0.681
FBG (mmol/L)	5.00 ± 0.72	5.06 ± 0.82	5.10 ± 0.87	5.15 ± 0.89	0.847
Scr (μmol/L)	69.22 ± 13.31	71.12 ± 16.23	70.38 ± 17.16	69.66 ± 15.58	0.948
UA (μmol/L)	343.58 ± 72.31	333.58 ± 87.98	352.57 ± 100.61	359.00 ± 89.26	0.245
ALB (g/L)	41.83 ± 3.35	42.45 ± 2.92	42.93 ± 3.27	42.09 ± 4.05	0.165
TC (mmol/L)	4.27 ± 0.94	4.32 ± 0.89	4.45 ± 0.93	4.58 ± 1.03	0.141
TG (mmol/L)	1.56 ± 1.34	1.51 ± 0.93	1.57 ± 0.71	1.85 ± 1.24	0.117
LDL-C (mmol/L)	2.47 ± 0.83	2.53 ± 0.78	2.63 ± 0.82	2.60 ± 0.87	0.475
HDL-C (mmol/L)	1.16 ± 0.27	1.13 ± 0.28	1.15 ± 0.28	1.15 ± 0.33	0.810
*Echocardiographic parameter*					
LAD (mm)	41.70 ± 4.71	42.19 ± 5.67	42.22 ± 5.37	43.80 ± 6.01	0.049
LVEF (%)	63.91 ± 5.58	62.62 ± 5.47	63.08 ± 5.33	61.75 ± 5.41	0.068
LVD (mm)	48.71 ± 4.02	49.08 ± 4.46	48.33 ± 4.08	48.76 ± 5.21	0.740
LVS (mm)	31.67 ± 3.78	32.40 ± 4.32	31.74 ± 3.58	32.31 ± 4.32	0.500
Ablation procedure					0.230
CPVI alone	58 (66.7)	59 (65.6)	64 (72.7)	51 (58.0)	
CPVI plus additional ablation	29 (33.3)	31 (34.4)	24 (27.3)	37 (42.0)	
Direct current cardioversion	22 (25.3)	26 (28.9)	26 (29.5)	29 (33.0)	0.740
*Medication*					
Oral anticoagulant					0.999
Warfarin	6 (6.9)	7 (7.8)	6 (6.8)	6 (6.8)	
Dabigatran	63 (72.4)	65 (72.2)	65 (73.9)	62 (70.5)	
Rivaroxaban	18 (20.7)	18 (20.0)	17 (23.3)	20 (22.7)	
ACEI/ARB	32 (36.8)	30 (33.3)	42 (47.7)	41 (46.6)	0.132
β-blocker	40 (46.0)	39 (43.3)	39 (44.3)	47 (53.4)	0.528
Amiodarone	58 (66.7)	62 (68.9)	62 (70.5)	67 (76.1)	0.557
Propafenone	14 (16.1)	16 (17.8)	10 (11.4)	12 (13.6)	0.643
*Biomarker*					
hs-CRP (mg/L)	1.90 ± 1.94	1.23 ± 1.26	1.88 ± 2.75	2.87 ± 3.54	0.001

All continuous variables are expressed as mean ± SD, and categorical variables are presented as count (percentage). BMI, body mass index; SBP, systolic blood pressure; DBP, Diastolic blood pressure; AF, atrial fibrillation; TIA, transient ischemic attack; FBG, fasting plasma glucose; Sc, serum creatinine; UA, uric acid; ALB, serum albumin; TC, total cholesterol; TG, triglyceride; LDL-C, low-density lipoprotein cholesterol; HDL-C, high-density lipoprotein cholesterol; ACEI, angiotensin-converting enzyme inhibitor; ARB, angiotensin receptor blocker; LAD, left atrium anteroposterior diameter; LVEDD, left ventricular end-diastolic diameter; LVESD, left ventricular end-systolic diameter; LVEF, left ventricular ejection fraction; hs-CRP, high-sensitivity C-reactive protein; CA-125, carbohydrate antigen-125.

Table 3 shows the number of events and the rates of recurrent AF for stratification according to CA-125 quartiles. The second (8.50–11.20 U/mL), third (11.21–14.65 U/mL), and highest quartiles of CA-125 (> 14.65 U/mL) were associated with higher incidence rates of AF recurrence than the first quartile (< 8.50 U/mL). There was a thresholding effect observed between CA-125 quartiles and recurrent AF. The rates of AF recurrence were the highest in the highest quartile, presenting a significant linear increasing trend (*P*-trend across quartiles < 0.001).

**Table 3:** Incidence of recurrent AF following RFCA based on CA-125 quartiles

Variable	CA-125 level				P trend
	Quartile 1	Quartile 2	Quartile 3	Quartile 4	
Quartile value (U/mL)	< 8.50	8.50–11.20	11.21–14.65	> 14.65	
Events/patients	10/87	12/90	19/88	44/88	
Incidence (%)	11.5%	13.3%	21.6%	50.0%	< 0.001

AF, atrial fibrillation; CA-125, carbohydrate antigen-125; RFCA, radiofrequency catheter ablation

**Fig. 4:**
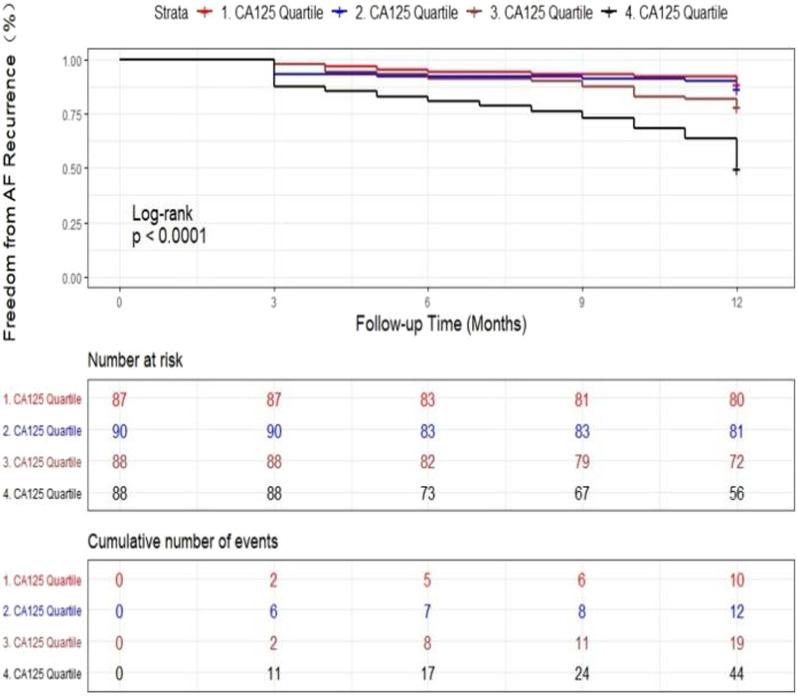
Kaplan–Meier curves of AF recurrence across CA-125 quartiles

Figure 4 indicates the cumulative incidence of AF recurrence according to CA-125 quartiles following the blanking period (log-rank *P* < 0.0001). In the fully adjusted model 3, the aHRs for those in the second, third, and highest quartiles of CA-125, compared to those in the first quartile (reference), were 1.085 (95% CI, 0.468–2.520; *P* = 0.849), 1.866 (95% CI, 0.867–4.019; *P* = 0.111), and 4.246 (95% CI, 2.113–8.533; *P* < 0.001), exhibiting a significant linear increasing trend (*P*-trend across quartiles < 0.001) (Table 4). Data on CA-125 analyzed as a continuous variable are indicated in Table 4. A per unit increase in Ln CA-125 was related to an aHR of 3.225 (95% CI, 2.258–4.606; *P* < 0.001).

**Table 4:** Hazard ratios of AF recurrence following RFCA based on CA-125 quartiles and continuous CA-125 levels

Variable	CA-125 quartile				*P*-trend	LnCA-125
	1	2	3	4		
Quartile value, U/mL	< 8.50	8.50–11.20	11.21–14.65	> 14.65		
Model 1	1.00 (Ref)	1.181 (0.510, 2.734)	1.981 (0.921, 4.261)	5.260 (2.645, 10.458)	< 0.001	3.670 (2.577, 5.226)
*P*-value		0.698	0.080	< 0.001		< 0.001
Model 2	1.00 (Ref)	1.181 (0.510, 2.734)	1.980 (0.920, 4.257)	5.185 (2.604, 10.325)	< 0.001	3.654 (2.561, 5.213)
*P*-value		0.697	0.081	< 0.001		< 0.001
Model 3	1.00 (Ref)	1.085 (0.468, 2.520)	1.866 (0.867, 4.019)	4.246 (2.113, 8.533)	< 0.001	3.225 (2.258,4.606)
*P*-value		0.849	0.111	< 0.001		< 0.001

CA-125, carbohydrate antigen-125

**Fig. 5:**
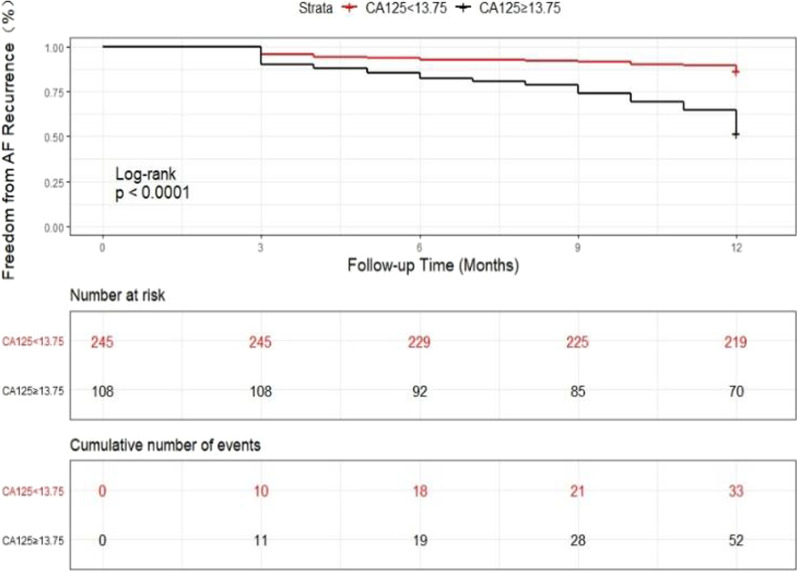
Kaplan–Meier curves of AF recurrence based on CA-125 levels below or above 13.75 U/mL

Patients with levels of CA-125 above the cut-off of 13.75 U/mL had higher rates of AF recurrence than those with CA-125 levels below 13.75 U/mL (33 patients [13.5%] vs. 52 patients [48.2%]; *P* < 0.001) (Table 5). Kaplan–Meier curves are depicted in Fig. 5. In the fully adjusted model 3, the risk of recurrent AF was higher in patients who had CA-125 levels above 13.75 U/mL than those with levels below the cut-off (aHR, 3.540; 95% CI, 2.268–5.525; *P* < 0.001) (Table 5).

**Table 5:** Hazard ratios of AF recurrence following RFCA based on CA-125 cut-off point of 13.75 U/mL

Variable	CA-125 level (U/mL)		*P*-value
	< 13.75	≥ 13.75	
Events/patients	33/245	52/108	
Incidence (%)	13.5%	48.2%	< 0.001
Model 1	1.00 (Ref)	4.178 (2.699, 6.468)	< 0.001
Model 2	1.00 (Ref)	4.125 (2.661, 6.397)	< 0.001
Model 3	1.00 (Ref)	3.540 (2.268, 5.525)	< 0.001

CA-125, carbohydrate antigen-125; RFCA, radiofrequency catheter ablation

### Relationship between the 12-month recurrence of AF following RFCA and risk factors

The univariate logistic regression analysis revealed that plasma CA-125, hs-CRP, LAD, PeAF, and intraoperative direct current cardioversion were significantly correlated with AF recurrence following RFCA (*P* < 0.001, Table 6). There was no multicollinearity between the above variables (VIF < 3). In addition, the multivariate logistic regression analysis demonstrated that hs-CRP (OR, 1.103; 95% CI: 1.005–1.210; *P* = 0.040), LAD (OR, 1.096; 95% CI, 1.042–1.153; *P* < 0.001), and plasma CA-125 (OR, 1.121; 95% CI: 1.075–1.169; *P* < 0.001) were independent risk factors of AF recurrence following RFCA (Table 6).

**Table 6:** Univariate and multivariate logistic regression analyses for predicting AF recurrence

Variable		Univariate			Multivariate	
	OR	95% CI	*P* value	OR	95% CI	*P* value
CA-125 (U/mL)	1.129	1.083–1.177	< 0.001	1.121	1.075–1.169	< 0.001
hs-CRP (mg/L)	1.145	1.050–1.248	0.002	1.103	1.005–1.210	0.040
LAD (mm)	1.108	1.055–1.162	< 0.001	1.096	1.042–1.153	< 0.001
LVEF (%)	0.960	0.917–1.004	0.077			
Persistent AF	2.731	1.628–4.579	< 0.001			
AF duration (months)	1.004	0.997–1.010	0.270			
Hypertension	1.207	0.731–1.993	0.461			
Diabetes	1.838	0.868–3.892	0.112			
Direct current cardioversion	2.359	1.415–3.933	0.001			
Age (years)	1.005	0.979–1.031	0.724			
Male	1.297	0.788–2.135	0.306			
BMI (kg/m^2^)	1.007	0.930–1.090	0.867			

CA-125, carbohydrate antigen-125; hs-CRP, high-sensitivity C-reactive protein; LAD, left atrium anteroposterior diameter; LVEF, left ventricular ejection fraction; AF, atrial fibrillation; BMI, body mass index

### Predictive value of CA-125 levels in the 12-month recurrence of AF following RFCA

**Fig. 6:**
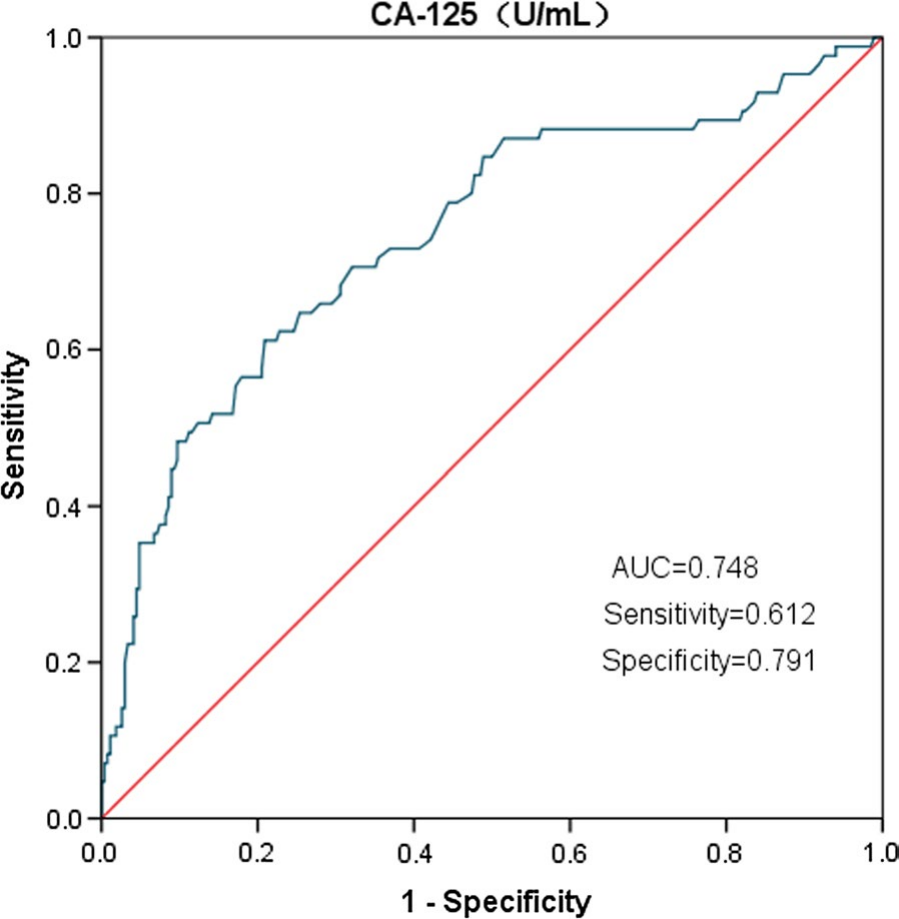
The ROC curve of CA-125

According to the ROC analysis (Fig. 6), the optimal cut-off point, specificity, sensitivity, positive predictive values, negative predictive values, and AUC values for CA-125 to predict recurrent AF were 13.75 U/mL, 79.1%, 61.2%, 48.1%, 86.5%, and 0.748 (95% CI, 0.683–0.812; *P* < 0.001), respectively. Kaplan–Meier analysis, conducted using the best cut-off point of CA-125 (13.75 U/mL), showed that the rate of 12-month AF recurrence was significantly higher in patients with CA-125 ≥ 13.75 U/mL than in those with CA-125 < 13.75 U/mL (Log-rank *P* < 0.0001, Fig. 5).

### Complications

After the RFCA, two patients underwent emergency pericardiocentesis due to pericardial tamponade. One patient had pseudoaneurysms. Four patients developed subcutaneous hematoma at the puncture site. One patient developed a transient ischemic attack. No patient developed stroke, myocardial infarction, atrial esophageal fistula, or pericarditis after RFCA. During the 12-month follow-up, two patients had pacemakers implanted due to sick sinus syndrome, and five patients developed gingival bleeding. No patient developed stroke or HF.

## Discussion

In this comprehensive analysis of patients with AF undergoing initial catheter ablation, elevated preoperative plasma CA-125 levels were related to a higher risk of recurrent AF. Following adjustment for known risk factors of recurrence, patients with CA-125 level in the highest quartile had a 4.246-fold higher risk of AF recurrence than those who had their CA-125 level in the first quartile. These findings were consistently observed when levels of CA-125 were analyzed as continuous data or at a cut-off value of 13.75 U/mL, indicating that the present results are robust and do not depend on a defined cut-off point. Adjustment for a wide range of potential confounders had little impact on the estimates of risk, indicating a lower probability of residual confounding. In the present study, further multiple regression analysis revealed that CA-125 levels (OR, 1.121; 95% CI: 1.075–1.169; *P* < 0.001) and hs-CRP levels (OR, 1.103; 95% CI: 1.005–1.210; *P* = 0.040) and LAD (OR, 1.096; 95% CI, 1.042–1.153; *P* < 0.001) were significantly associated with the prognosis of AF.

Dudink et al. [16] compared blood samples from 60 idiopathic AF patients with samples from 120 healthy patients in sinus rhythm (SR) and found that CA-125 levels are significantly higher in idiopathic AF patients than in SR controls. Multivariate analysis showed that CA-125 level (OR, 1.68; 95% CI: 1.07–2.64; *P* = 0.026) was independently associated idiopathic AF. It revealed that CA-125 could be a marker for the occurrence of AF in patients without structural heart disease. In a study by Sekiguchi et al*.* [17], of 746 menopausal women with a 10-year follow-up, baseline CA-125 levels were significantly higher in the AF group than the group without AF (11.4 ± 6.3 U/mL vs. 7.7 ± 3.2 U/mL; *P* < 0.01), and multiple Cox regression analysis showed that CA-125 level (HR, 1.29; 95% CI: 1.10–1.51; *P* = 0.02) is an independent predictor of AF occurrence. Higher CA-125 levels were associated with new-onset AF in healthy postmenopausal women. Yucel et al*.* [11] prospectively studied 149 patients with systolic HF in SR. At 22.1 ± 11.0 months follow-up, 36 patients (24.2%) developed new-onset AF, and baseline CA-125 levels were significantly higher in these AF patients than the maintained SR controls (*P* = 0.001). Multiple regression analysis showed that CA-125 levels are independently associated with an increased risk of new-onset AF in patients with systolic HF. In a study that included 205 patients with stable systolic HF, Kaya et al*.* [18] found that CA-125 levels are significantly higher in the AF group than in the SR control, with a statistically significant difference. Multifactorial analysis showed that CA-125 levels could be used to predict the presence of AF in systolic HF patients. These results indicate that CA-125 may act as a biomarker for AF. However, the underlying pathophysiological mechanism of elevated plasma CA-125 levels in AF was still under debate. Several studies have suggested that the causes might be as follows: (I) AF leads to various hemodynamic disorders by causing left ventricle systolic and diastolic dysfunction. These dysfunctions increase mechanical stress on mesothelial cells, and AF could be accompanied by the inflammatory response [19]. Inflammatory stimulus and abnormal mechanical stress are transmitted to the cytoplasm via c-Jun N-terminal kinase pathways and induce CA-125 synthesis by the mesothelial cells [9]. In addition, changes in the morphocytology and stability of the cell membrane activate the extracellular domain of CA-125 to be shed from the mesothelial cells [9]. (II) CA-125 might act as a secondary cytokine [20]. Elevation of inflammatory biomarkers, such as interleukin-6, C-reactive protein, and tumor necrosis factor-α, had been observed in patients with AF [21], and an in vitro study had found that inflammatory factors, such as tumor necrosis factor-α, could promote the secretion of CA-125 [22, 23]. (III) The occurrence and development of AF are accompanied by myocardial remodeling [24]. Based on present developments in molecular biology, it is clear that myocardial remodeling results in pathological cardiomyocyte hypertrophy with embryonic gene re-expression. During this process, proto-oncogenes are activated, which stimulate growth factors present in the embryonic heart, leading to increased CA-125 levels [25, 26]. All the above reports indicate that plasma CA-125 levels are highly correlated with the occurrence and development of AF.

We do not consider that increased CA-125 levels mainly result in the development of AF, but rather that the electrophysiological, structural, and hormonal factors that lead to the development of AF seem to ultimately lead to elevated CA-125 levels. Overall, we hypothesize that plasma CA-125 levels could reflect myocardial remodeling and inflammatory status in AF patients, but this hypothesis warrants confirmation. Our hypothesis is supported based on our results that higher CA-125 quartiles were associated with higher levels of hs-CRP and LAD and that the differences across CA-125 quartiles were significant (*P* < 0.05). Furthermore, CA-125 levels were positively correlated with LAD (r = 0.144, *P* = 0.007). Our results are consistent with the results of Kaya et al*.* [18]. Although the difference in LVEF across CA-125 quartiles was not significant, correlation analysis showed that there was a significant although weak negative correlation between CA-125 level and LVEF (r =  − 0.108, *P* = 0.044). The results are supported by the results of Yilmaz et al*.* [27]*.* However, there was no significant correlation between hs-CRP level and CA-125 level (r = 0.073, *P* = 0.172). The potential reason may be that CA-125 is able to reflect more organism information besides the inflammatory status in AF patients than hs-CRP. Myocardial remodeling and inflammation are closely correlated with the occurrence, development, and recurrence of AF. Meanwhile, the present study showed PeAF is associated with higher levels of CA-125 than PAF (Fig. 2), and the duration of AF was significantly longer in sequence with increasing CA-125 quartiles, which is consistent with the report by Arbault-Biton et al. [28], considering that this may be attributed to the more severe myocardial remodeling and inflammatory conditions in the longer-duration AF than in the shorter-duration AF. In the present study, increasing pre-interventional CA-125 levels were found to be closely associated with a higher risk of AF recurrence and were an independent risk factor for postoperative AF recurrence. Our results are supported by the results of Huang et al. [6]. However, in their study, the optimal cutoff value for plasma CA-125 to predict AF recurrence following ablation was 11.05 U/mL, which was less than the 13.75 U/mL in the present study. Potential reasons for this may include, firstly a greater proportion of female patients in our study (43.6% vs. 35.1%), secondly a longer duration of AF in the recurrence group of our study (29.12 ± 33.32 months vs. 27.79 ± 37.27 months and finally, in the study performed by Huang et al., up to 67.5% of patients were receiving long-term statin therapy. Statins have an anti-inflammatory effect [17], which may have resulted in lower CA-125 levels.

AF recurrence could be predicted by measuring plasma CA-125 levels, and the sensitivity, specificity, positive predictive value, and negative predictive value of plasma CA-125 in predicting postoperative AF recurrence were 61.2%, 79.1%, 48.1%, and 86.5%, respectively. These suggest that patients with baseline CA-125 levels below 13.75 U/mL, who we selected for ablation, had a lower probability of post-procedure AF recurrence and greater patient benefit.

As a biomarker, CA-125 exhibits multiple theoretical advantages. Firstly, compared with other biomarkers, such as brain natriuretic peptide, CA-125 is more stable and has a longer half-life varying from 5.1 to 12 days, providing greater reliability over time [29]. Secondly, plasma CA-125 levels are not significantly affected by sex, age, BMI, and renal function [6]. Finally, the assay for CA-125 is a standardized and highly reproducible assay that is simple, without the need for resting conditions, and relatively cheap. Thus, plasma CA-125 is an advantageous and potential biomarker for predicting AF recurrence.

Hs-CRP, an acute-phase protein, is a reliable marker of systemic inflammation. Inflammatory mediators released in response to inflammation can play an important role in the development and progression of AF by causing electrical and structural remodeling of the atrium [30]. Elevated levels of inflammatory biomarkers such as hs-CRP and IL-6 have been observed in patients with paroxysmal and persistent AF [21], suggesting an inextricable link between AF and inflammation. The present study showed a significant difference in baseline hs-CRP levels between the AF recurrence and nonrecurrence groups (2.76 ± 3.45 mg/L vs. 1.71 ± 2.18 mg/L, *P* = 0.004), and further multivariate regression analysis showed that hs-CRP was an independent risk factor for postoperative AF recurrence. This is consistent with the results of a recently conducted prospective study by Meyre et al. [31] that enrolled 711 AF patients; in their study, elevated pre-ablation hs-CRP levels were strongly associated with a higher risk of AF recurrence.

Electrical reconduction of one or more PVs may play an important role in the late recurrence of AF [32]. In patients with severe left atrial remodeling, the CPVI line is longer and catheter manipulation is more difficult. Therefore, these patients may have more frequent electrical reconduction of the PVs. Left atrial enlargement is related to atrial remodeling, which is associated with recurrent AF following RFCA [33]. LAD is widely used in research and clinical practice as a non-invasive approach to measure left atrial enlargement. Berruezo et al*.* [34] found that LAD can independently predict AF recurrence, and that an increase in LAD was linearly associated with the risk of AF recurrence. In line with our findings, the association between left atrial enlargement and AF recurrence has also been demonstrated by many others.

### Limitations

This study has some potential limitations. Firstly, it had a small sample size and is a single-center study. Secondly, in this study, routine follow-up was performed with 12-lead ECG or 24-h Holter monitors, which are less accurate than the 7-day dynamic electrocardiogram and may lead to ascertainment bias. Finally, we determined plasma CA-125 levels only at baseline, and therefore, we were unable to assess the effects of changes in CA-125 levels over time or the effect of postoperative CA-125 levels.

## Conclusions

Elevated CA-125 levels determined prior to RFCA are related to a higher risk of AF recurrence and are an independent predictor of 12-month AF recurrence after RFCA. CA-125 is an important easily available and inexpensive biomarker for predicting AF recurrence. We consider that patients with increasing pre-interventional CA-125 levels must be closely followed up.

## Data Availability

The figure and table data used to support the findings of this study are included within the article.

## Abbreviations

CA-125: Carbohydrate antigen-125
AF: Atrial fibrillation
HF: Heart failure
RFCA: Radiofrequency catheter ablation
aHR: Adjusted hazard ratio
LAD: Left atrium anteroposterior diameter
PeAF: Persistent atrial fibrillation
hs-CRP: High-sensitivity C-reactive protein
LVEF: Left ventricular ejection fraction
ECG: Electrocardiogram
BMI: Body mass index
SBP: Systolic blood pressure
DBP: Diastolic blood pressure
TIA: Transient ischemic attack
FBG: Fasting plasma glucose
Sc: Serum creatinine
UA: Uric acid
ALB: Serum albumin
TC: Total cholesterol
TG: Triglyceride
LDL-C: Low-density lipoprotein cholesterol
HDL-C: High-density lipoprotein cholesterol
ACEI: Angiotensin-converting enzyme inhibitor
ARB: Angiotensin receptor blocker
LVEDD: Left ventricular end-diastolic diameter
LVESD: Left ventricular end-systolic diameter
CPVI: Circumferential pulmonary vein isolation
ROC: Receiver operating characteristic
AUC: Area under the curve
SR: Sinus rhythm
